# Adrenal metastasis as the initial diagnosis of synchronous papillary and follicular thyroid cancer

**DOI:** 10.1186/s40842-020-00109-0

**Published:** 2020-11-04

**Authors:** Xin He, Scott A. Soleimanpour, Gregory A. Clines

**Affiliations:** 1 Department of Internal MedicineDivision of MetabolismEndocrinology & Diabetes, University of Michigan, 1500 East Medical Center Drive, Ann Arbor, MI 48109 USA; 2 Veterans Affairs Ann Arbor Healthcare System, 2215 Fuller Road, Ann Arbor, MI 48105 USA

**Keywords:** Differentiated thyroid cancer, Thyroid cancer metastasis, Metastases to adrenal glands

## Abstract

**Background:**

Differentiated thyroid cancer uncommonly presents with distant metastases. Adrenal metastasis from differentiated thyroid cancer presenting as the initial finding is even less common.

**Case Presentation:**

A 71-year-old male was incidentally found on chest CT to have bilateral thyroid nodules, which were confirmed on ultrasound. Fine needle aspiration of the dominant right 3.3 cm nodule contained histologic features most consistent with Bethesda classification III, and repeat fine needle aspiration revealed pathology consistent with Bethesda classification II. Follow-up thyroid ultrasound showed 1% increase and 14% increase in nodule volume at one and two years, respectively, compared to baseline. Prior to the second annual thyroid ultrasound, the patient was incidentally found to have a 4.1 cm heterogeneously enhancing mass in the right adrenal gland on CT of the abdomen and pelvis. Biochemical evaluation was unremarkable with the exception of morning cortisol of 3.2 µg/dL after dexamethasone suppression. The patient then underwent laparoscopic right adrenal gland excision, which revealed metastatic follicular thyroid carcinoma. Total thyroidectomy was then performed, with pathology showing a 4.8 cm well-differentiated follicular thyroid carcinoma of the right lobe, a 0.5 cm noninvasive follicular thyroid neoplasm with papillary-like nuclear features of the left lobe, and a 0.1 cm papillary microcarcinoma of the left lobe. Thyrotropin-stimulated whole body scan showed normal physiologic uptake of the remnant thyroid tissue without evidence of other iodine avid disease. The patient then received radioactive iodine. At follow-up 14 months after total thyroidectomy, he remains free of recurrent disease.

**Conclusion:**

Despite following the recommended protocol for evaluation and surveillance of thyroid nodules, thyroid cancer can be challenging to diagnose, and may not be diagnosed until distant metastases are identified.

## Background

Thyroid cancer is the most common endocrine malignancy and twelfth most common cancer in the United States [1]. Differentiated thyroid carcinomas (DTC) account for the vast majority of all thyroid cancer cases, of which roughly 80% are papillary thyroid carcinoma (PTC) and 10–15% are follicular thyroid carcinoma (FTC) [2],medullary and anaplastic thyroid cancer make up the remaining 5% of cases. The concurrent presentation of multiple histologic types of thyroid cancer in the same patient is rare, accounting for approximately 0.06% of thyroid cancers in database analyses [3]. Amongst DTC, prognosis is excellent for most patients, even for the 20–50% of cases that present with local metastases [4]. However, more aggressive disease can be seen; approximately 2–3% of DTC cases present with distant metastases on diagnosis [5, 6]. We report a case of DTC manifesting with both PTC and FTC, which was diagnosed on evaluation of adrenal metastasis.

## Case presentation

**Fig. 1:**
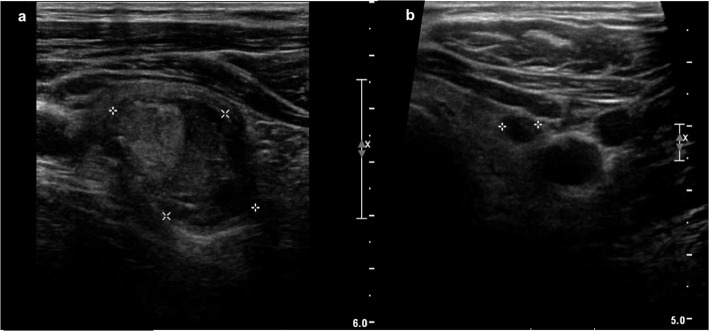
Thyroid ultrasound. **a**. Dominant 3.3 × 2.2 × 1.8 cm right thyroid nodule with heterogeneous echogenicity, mixed solid and cystic composition, lobulated borders, without echogenic foci, and without increased vascularity. **b**. One of several sub-centimeter left thyroid nodules that were hypoechoic, cystic, wider than tall, with smooth margins, without echogenic foci, and without increased vascularity

**Fig. 2:**
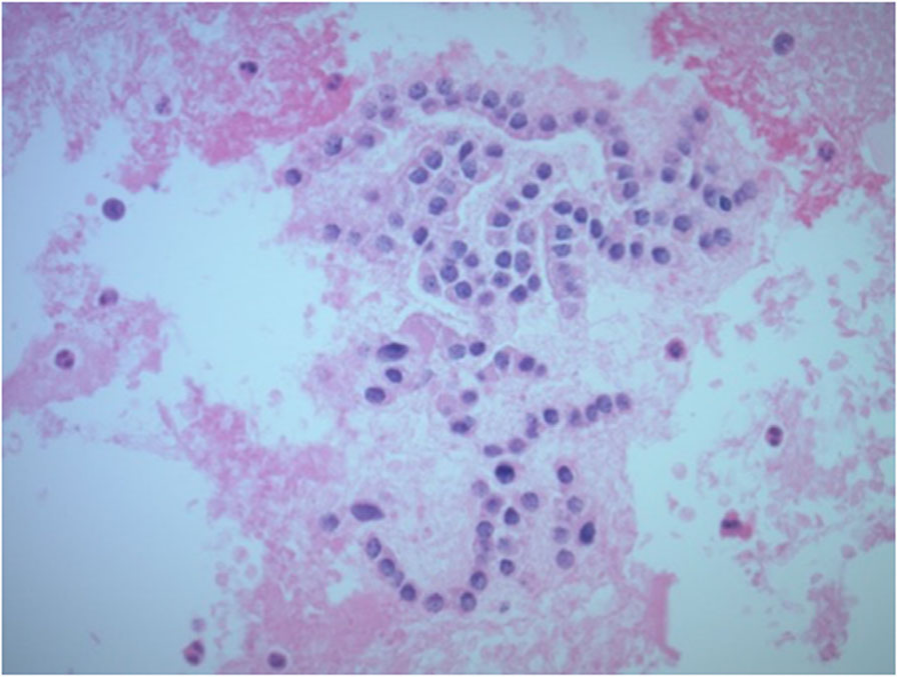
Fine needle aspiration of dominant right thyroid nodule: hypercellular aspirate with follicular epithelial cells in sheets and microfollicles, some with Hurthle cell changes and colloid in the background, without significant atypia (hematoxylin–eosin stain, × 400)

A 71-year-old male with past medical history of hypertension, type 2 diabetes, stage 3 chronic kidney disease, kidney stones, intraductal papillary mucinous neoplasm status post distal pancreatectomy, and 50-pack-year smoking history, was incidentally found on surveillance chest CT of pulmonary nodules to have bilateral thyroid nodules. Thyroid ultrasound confirmed a dominant 3.3 × 2.2 × 1.8 cm right thyroid nodule with heterogeneous echogenicity, mixed solid and cystic composition, lobulated borders, without echogenic foci, and without increased vascularity (Fig. 1a). Multiple sub-centimeter left thyroid nodules that were hypoechoic, cystic, wider than tall, with smooth margins, without echogenic foci, and without increased vascularity, were also noted (Fig. 1b). No lymphadenopathy in the neck was identified. Fine needle aspiration (FNA) of the dominant right thyroid nodule reported a hypercellular aspirate with follicular epithelial cells in sheets and microfollicles, some with Hurthle cell changes and colloid in the background, without significant atypia (Fig. 2). These features were considered to be most consistent with Bethesda classification III, follicular lesion of undetermined significance (FLUS). Repeat FNA of the same nodule three months later showed benign appearing follicular epithelial cells in few microfollicles groups and many with Hurthle cell changes amid scant colloid, consistent with a benign colloid nodule, Bethesda classification II. Molecular testing was not completed with either FNA. Follow-up thyroid ultrasound completed annually for the next two years showed gradual growth of the right dominant nodule to 3.2 × 2.3 × 1.8 cm at one year (1% increase in volume) and 3.3 × 2.5 × 1.8 cm at two years (14% increase in volume compared to baseline); the smaller left nodules remained sub-centimeter in size without interval change in ultrasound features.

**Fig. 3:**
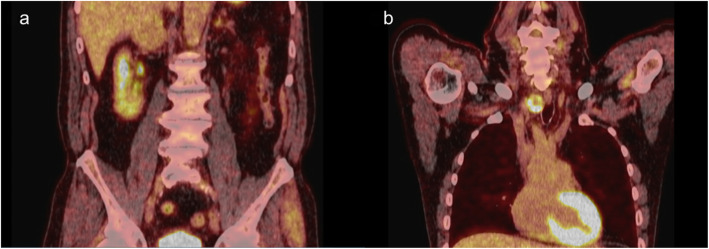
^18^F-FDG PET/CT: **a**. The right adrenal mass has mild homogeneous activity (maximum SUV of 2.7), which is similar to the activity to the liver. **b**. Posterior aspect of the right lobe of the thyroid gland is primarily peripherally FDG avid (maximum SUV of 12.7)

**Fig. 4:**
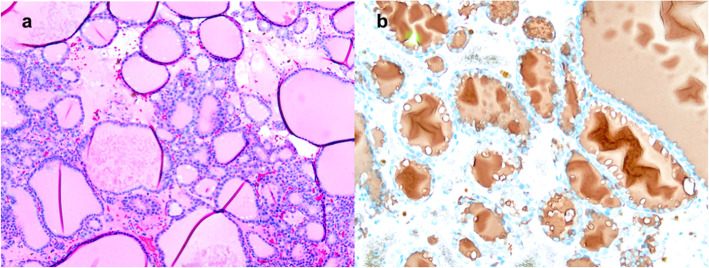
**a**. Right adrenal gland surgical pathology: metastatic follicular thyroid carcinoma (hematoxylin–eosin stain, × 100). **b**. Right adrenal gland surgical pathology: metastatic follicular thyroid carcinoma with positive thyroglobulin stain (thyroglobulin immunostain, × 100)

Two months prior to the second annual thyroid ultrasound, the patient underwent CT of the abdomen and pelvis for evaluation of new onset microscopic hematuria and was incidentally noted to have a 4.1 cm heterogeneously enhancing mass in the right adrenal gland. No enlarged lymph nodes were observed. In retrospect, this had grown from the heterogeneous 1.8 cm nodule on imaging completed two years prior (on the same CT that first identified the thyroid nodules, though the adrenal finding was not reported at the time) and new when compared to imaging nine years prior. On whole body ^18^F-FDG PET/CT, the adrenal nodule had minimal FDG avidity (maximum SUV of 2.7) (Fig. 3a), but the right thyroid nodule had high peripheral FDG avidity (maximum SUV of 12.7) (Fig. 3b). Biochemical evaluation was negative for primary aldosteronism (plasma renin activity 13.7 ng/mL/hr, aldosterone 18.4 ng/dL) and pheochromocytoma (plasma metanephrines 0.24 nmol/L, plasma normetanephrines 18.4 ng/dL). Overnight 1 mg dexamethasone suppression resulted in a borderline morning cortisol of 3.2 µg/dL, but late night salivary cortisol was measured twice thereafter and within normal range, 0.055 µg/dL and 0.060 µg/dL; 24-h urinary free cortisol testing was not completed. The patient did not have any clinical signs or symptoms suggestive of cortisol excess. The patient then underwent laparoscopic right adrenal gland excision due to concern for malignancy. Pathology revealed metastatic follicular thyroid carcinoma, which stained positive for thyroglobulin (Figs. 4a and 4b).

**Fig. 5:**
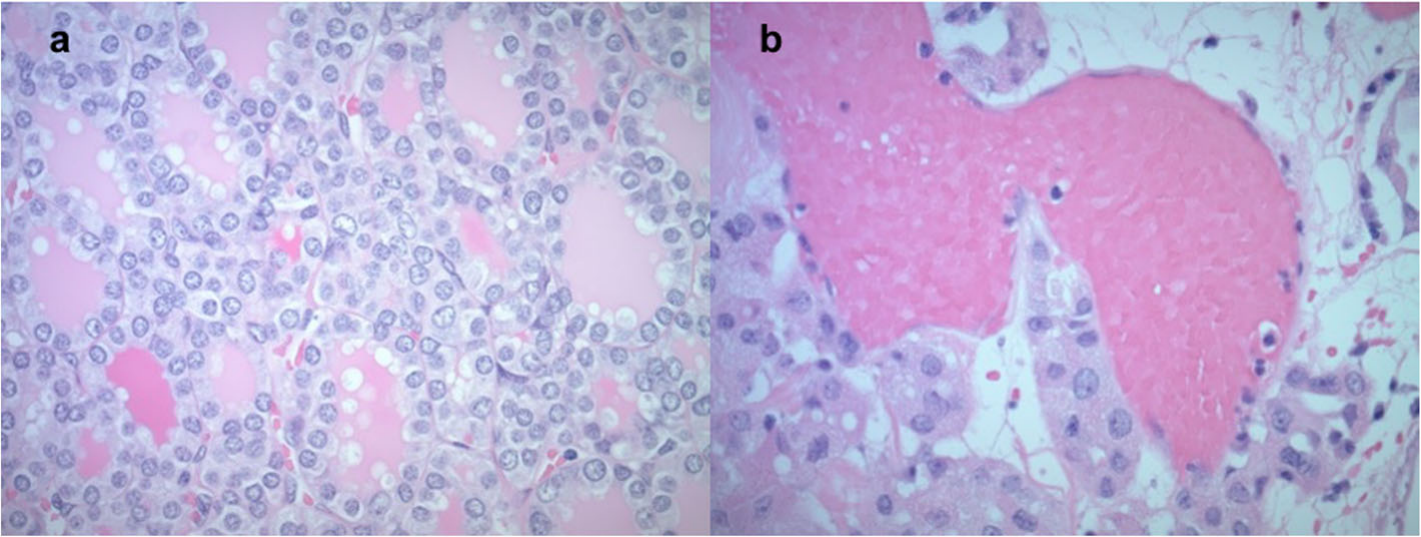
Right thyroid lobe surgical pathology: Follicular carcinoma of the right lobe (**a**), well-differentiated 4.8 cm with capsular invasion and probable lymphovascular invasion (**b**), confined to thyroid (hematoxylin–eosin stain, × 400)

**Fig. 6:**
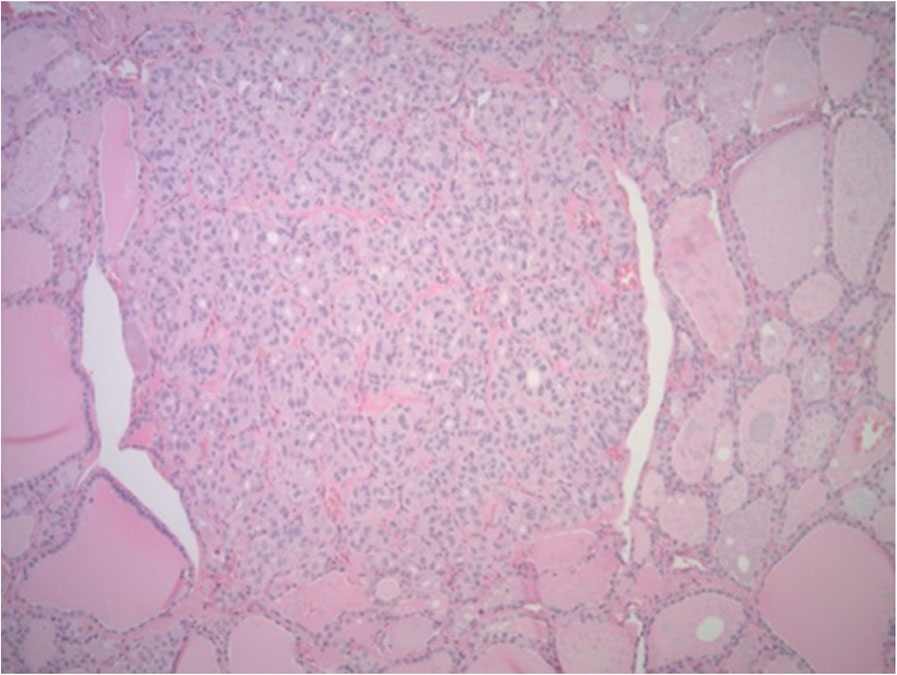
Left thyroid lobe surgical pathology: Papillary microcarcinoma of the left lobe 0.1 cm, confined to the thyroid with negative margins, and with background multinodular hyperplasia (hematoxylin–eosin stain, × 40)

Total thyroidectomy was then performed. Pathologic evaluation reported a 4.8 cm well-differentiated FTC of the right lobe (Figs. 5a and b), a 0.5 cm noninvasive follicular thyroid neoplasm with papillary-like nuclear features (NIFTP) of the left lobe, and a 0.1 cm papillary microcarcinoma of the left lobe with negative margins (Fig. 6). Background multinodular hyperplasia and lymphatic invasion were observed, but the margins were negative and no extra-thyroidal extension noted; lymph node dissection was not completed. Serum thyroglobulin was 7.8 ng/mL seven weeks after total thyroidectomy. Thyrotropin-stimulated ^131^I whole body scan reported normal physiologic uptake of the remnant thyroid tissue without evidence of other iodine avid disease. The patient then received 106.8 mCi of ^131^I, and post-radioactive ^131^I whole body scan revealed no new foci of iodine avid disease. Eight months after total thyroidectomy, serum thyroglobulin decreased to 0.1 ng/mL with negative thyroglobulin antibodies. At follow-up 14 months after total thyroidectomy, the patient’s thyroglobulin level remained low, 0.1 ng/mL, and he was continued on suppressive levothyroxine treatment with thyroid stimulating hormone level of 0.019 uIU/mL.

## Discussion and conclusions

Multiple features of this patient case were unusual. Firstly, the diagnosis of thyroid malignancy was not made until the patient presented with a rare location for distant metastases. Second, the patient underwent multiple fine needle aspirations of the dominant right nodule, ultimately yielding reassuring pathology, and surveillance imaging showed stability for years—yet the patient was later found to have thyroid cancer. Finally, the patient was found to have different histologic types of thyroid malignancy in each thyroid lobe.

Only 2–3% of DTC cases present with distant metastases on diagnosis [5, 6]. Risk factors for presentation with distant metastases include male sex, age greater than 45, non-Caucasian race, tumor size greater than 40 mm, and FTC. Male sex, older age, tumor size, and FTC diagnosis were relevant risk factors in our patient. In FTC specifically, the most common sites of distant metastases are bone (42%), lung (33%), and brain (17%) [7], which have been attributed primarily to hematogenous spread [8]. Adrenal metastases are rare, accounting for approximately 1.7% of DTC distant metastases [9], with only seven cases of FTC with metastases to the adrenal gland reported in the literature [9–15]. In four cases, the diagnoses of adrenal gland metastases were made during the initial workup following diagnosis of FTC, while the other three diagnoses were made during FTC surveillance, 3–12 years after initial diagnosis. Of the six cases reporting the imaging modality that identified the adrenal metastases, four cases employed ^131^I whole body scan alone, and two used ^131^I-SPECT/CT. Of note, one adrenal lesion was negative on ^131^I whole body scan, but was identified on CT imaging. Only one case also utilized ^18^F-FDG PET/CT, which revealed a high maximum SUV of 9.5 in the adrenal lesion. Three cases were treated with radioactive iodine, three underwent adrenalectomy, and one treated with gamma knife radiosurgery. Of the five cases reporting clinical outcome at the time of publication, all but one case reported the patients were alive.

Compared to prior reports, our case is unique in its clinical course, with the identification of the adrenal gland metastasis leading to the diagnosis of FTC, rather than the reverse. This is also only the second case to report lack of uptake of the adrenal metastasis on the ^131^I whole body scan. In contrast to the one other case that utilized ^18^F-FDG PET/CT, the adrenal metastasis in our case did not have high FDG avidity.

Another unusual feature of this case was the patient’s initial presentation of incidental thyroid nodules. It is notable that the first FNA result had features felt to be consistent with FLUS, prompting repeat aspiration, which resulted as a benign colloid nodule. It is possible the benign nodule transformed into a carcinoma following FNA. An estimated 2% of thyroid malignancies arise from preexisting benign thyroid nodules, and analyses of tumor markers in thyroid nodules suggest that some follicular and Hurthle cell adenomas may be precursors to carcinomas [16]. Thus, perhaps our patient’s nodule was, in fact, a follicular adenoma. It is also possible the benign FNA result was a false negative. Retrospective studies suggest that false negative FNA cases occur 4–6% of the time and can be attributed to both sampling error [17], as well as interpretation error in nearly equal proportion [18]. While intuitively larger nodules may be associated with greater sampling error [19, 20], other data suggests this has not been consistently observed [21, 22]. With respect to interpretation error, literature suggests follicular neoplasms are more commonly misinterpreted than non-follicular lesions, the former making up 79% of interpretation error cases [18]. In this patient, risk factors for potentially a false negative FNA include the thyroid nodule size as well as presence of follicular neoplasm.

Our patient was ultimately diagnosed with FTC of the right lobe, NIFTP of the left lobe, and papillary microcarcinoma of the left lobe. While not as exceptional as reports of concurrent presentation of DTC with medullary or anaplastic thyroid carcinoma [23–25], the presence of multiple subtypes of thyroid carcinoma in the same patient is rare [3]. The simultaneous occurrence of both follicular and papillary neoplasms can be attributed to their common cellular origin from follicular epithelial thyroid cells involved in iodine metabolism [26].

This case is unexpected in both its presentation as well as the ultimate diagnosis. It demonstrates that the diagnosis of DTC can be challenging, even in the setting of following the recommended protocol for evaluation and surveillance of thyroid nodules.

## Data Availability

Not applicable.
